# Supplementary material for: Are carbon emissions trading and green financial instruments synergistic? -Comprehensive quantitative research based on content analysis
Source: PLoS One. 2024 Mar 7;19(3):e0298601. doi: 10.1371/journal.pone.0298601 (PMC10919601; doi:10.1371/journal.pone.0298601)
Supplement: S1 Data — (ZIP) [file pone.0298601.s001.zip › Data/地级市Z_F工作报告.zip!/╜¡╦╒/─╧═¿╩╨/└┤╫╘╣1⁄2╓┌║┼í╛┬φ┐╦╩2╛▌═°í┐_2016.docx]

各位代表：

　　现在，我代表市人民政府向大会作工作报告，请予审议，并请市政协委员和其他列席人员提出意见。

　　一、2015年和“十二五”回顾

　　过去的一年，我们全面贯彻党的十八大和十八届三中、四中、五中全会精神，认真落实习近平总书记视察江苏时的重要讲话精神，在省委、省政府和市委的正确领导下，扎实推进“提质、扩量、增效、惠民”，较好完成全年目标任务。预计，地区生产总值6100亿元，增长9.5%；一般公共预算收入625.6亿元，增长13.8%；固定资产投资4376亿元，增长12.3%；社会消费品零售总额2380亿元，增长10%；进出口总额315.8亿美元，其中出口228.3亿美元、增长1.5%；注册外资实际到账25.8亿美元，与去年持平；城镇居民人均可支配收入36440元，农村居民人均可支配收入17400元，分别增长9.2%和10%；居民消费价格涨幅1.8%；城镇登记失业率1.92%；全社会研发投入占GDP比重2.55%；完成省下达节能减排任务。

　　（一）经济在转型升级中保持平稳健康发展。坚定不移采取一系列针对性政策措施，打好稳增长攻坚战。主导产业、新兴产业齐头并进。颁布实施《中国制造2025南通纲要》，规模以上工业总产值达1.38万亿元，工业技改投入占比68.6%。主导产业智能化趋势明显，涌现出一批“两化融合”示范试点企业。成为省级建筑产业现代化示范城市。新兴产业产值4552亿元，智能装备、生物医药、新材料等产业加快集聚。服务业应税销售增长20%，现代物流、科技、旅游等服务业和楼宇经济、平台经济快速发展，省级旅游度假区达4个。新业态、新模式加速涌现。全市宽带IP城域网出口带宽达2130G，互联网国际通信专用通道开通，智慧生活、教育体验馆等智慧南通示范项目建成。海工船舶、装备制造服务化进程加快，航天航空、工业机器人、智慧建筑等种子产业培育取得进展。云计算、大数据、跨境电子商务、知识流程外包、供应链管理等新业态加快成长，家纺等领域众创、众包、众筹模式发展势头良好。大众创业、万众创新蓬勃兴起。高新技术产业产值6203亿元，高新技术企业和省级以上研发机构分别达750家、559家。以南通高新区、产业技术研究院为龙头的创新创业载体建设成效明显，建成市级以上示范性众创空间9家，省级以上产业技术创新联盟达16家，“创新南通”、创业融资服务等平台上线运行。出台10多个支持科技创新的政策意见，发行“通创币”，举办“通创荟”、“江海英才创业周”等系列活动，国家“千人计划”人才达120名、省“双创”人才达211名，创投、股权投资机构达160家。南通大学顾晓松教授当选中国工程院院士。

　　（二）全面深化改革蹄疾步稳。各项国家级、省级改革试点有序实施。行政体制改革深化。开展行政审批制度改革“五大整治行动”。国家级相对集中行政许可权、综合行政执法体制改革试点取得突破，南通市及各县（市）区行政审批局成立，“一枚印章管审批”初步实现。企业注册登记“单一窗口”、“一照一码”实施到位。财税金融及土地等要素改革加快。财政预算管理、专项资金整合使用有效加强。各项贷款余额6081亿元，债券融资333亿元，股权融资166亿元，银行业金融机构增至40家，上市企业、“新三板”挂牌企业分别达32家、39家。陆海统筹土地管理创新、开发性金融促进海洋经济发展取得重要成果。国资国企、户籍制度、价格机制、农村产权、不动产登记等改革有序开展。社会和生态领域改革加强。教育领域综合改革统筹推进，联合办学、中考改革深入实施。综合医改持续深化，公立医院医药价格改革全面实施，分级诊疗体系加快构建。省级足球改革发展试点启动。养老服务公建民营等试点取得进展。食品药品重点领域监管机制不断完善。生态补偿、环保准入、排污权交易、环境污染责任保险、资源环境责任审计等制度加快建立。

　　（三）陆海统筹江海联动开放开发成效明显。强化特色、融合发展，区域开放开发空间进一步拓展。对接国家战略取得突破。对“一带一路”沿线国家和地区投资增长42.5%，中奥、中意生态园建设初显成效。对接上海自贸区取得新进展，在投资贸易便利化、金融等方面复制推广一批体制机制创新经验，市场采购贸易方式试点全面运行。与长江经济带重点城市合作持续深化。公铁水、江海河联运体系加快构建，沪通长江大桥快速建设，宁启铁路复线电气化改造试运营，海启高速开工。洋口港、启东港口岸开放和如皋港保税物流中心通过国家验收，兴东机场开通国际航班、年旅客吞吐量116万人次。港产城融合发展。南通港总体规划修编形成初步成果，通海港区启动建设，通州湾港区总体规划通过评审，洋口、吕四港区航道项目积极实施。沿海前沿区域高端装备、新材料、物流等特色产业基地（园区）建设快速推进，“区镇合一”重点镇综合配套功能增强。通州湾江海联动开发示范区获国家发改委同意、省政府批准设立。陆海生态统筹保护。落实生态红线区域保护规划，国家生态文明先行示范区和国家海洋生态文明建设示范区获批。实施212个节能及循环经济项目、44个重点污染减排项目，一批环境基础设施建成运行。新增造林面积全省第一。大气、水污染防治深入开展，PM2.5平均浓度降幅和空气质量良好天数全省领先，成为省大气污染防治工作优秀城市和省节水型城市。

　　（四）以新型城镇化促进城乡发展一体化取得新进展。发挥规划对城乡统筹发展的引领作用，形成城市“多规合一”初步成果。中心城市功能持续完善。381个城建项目完成投资178亿元，市区组团连接和重点片区交通进一步优化。新城区商业商务功能增强，观音山新城配套建设加快，市北新城重点功能板块特色显现，南部新城形象不断提升，一批连接通州区和主城区的骨干道路建成通车。“两河两岸”绿廊建设快速实施，星岛水岸区域功能性项目加快集聚。唐闸等历史文化保护项目有序推进，南通·1895园区获批海峡两岸文创产业合作区。建设国家现代农业示范区开局良好。农业现代化水平保持苏中苏北第一。粮食生产实现“十二连增”，设施农（渔）业达174万亩，高标准农田达377万亩。现代农（渔）业园区、农产品加工集中区建设加强。“全托管”经营主体达1145家，建成省级以上龙头企业77家、省级示范家庭农场28家。南通农产品特色馆等电商平台上线交易。国家农业科技园区获批建设。城乡环境整治深入实施。市容环境整治和市区水环境整治任务全面完成，获批省优秀管理城市。启动农村环境“四位一体”长效管理升级版建设，河道清障管护等工作取得突破，“美丽乡村”、“水美乡村”建设试点有力展开。

　　（五）社会建设和治理力度加大。推动民生改善与经济发展互动并进，一般公共预算支出中民生投入比重达75%。民生保障扎实有效。新增就业8.24万人，扶持创业1.31万人。城乡居民基本养老保险制度进一步完善，职工、居民医疗保险分别实现市级、城乡统筹，企业退休人员养老金、居民基础养老金稳步提高。每千名老人养老床位数达31.5张，居家养老、医养结合等协调发展。市区安置动迁户1.2万户。公共服务和产品更加丰富。教育现代化示范区建设有力推进，基础教育质量持续提升，职业教育产教融合进程加快，高等教育国际合作取得新进展。基层医疗卫生服务机构标准化建设深入实施，医联体建设和卫生接轨上海成效显现。唐闸近代工业遗存“申遗”通过国家文物局专家评审，一批文体重大项目和设施加快布局，成为省级公共体育服务体系示范区。全面依法治市积极推进。获得地方立法权，启动地方性法规和政府规章制订工作。深入开展法治创建活动，基层民主建设持续加强，政社互动实现街道（镇）全覆盖，92%的城市社区、85%的农村社区达到省级和谐社区建设标准。“一综多专”大调解、社会治安防控、综治基层基础、安全生产、应急管理体系进一步完善。民族、宗教和军民融合、国家安全等工作不断加强。

　　各位代表，一年来，我们认真开展“三严三实”专题教育，坚持领导带头、以上率下，自觉把“三严三实”要求体现到政府自身建设的各个方面。强化服务政府建设，完成政府机构改革任务，四级政务服务体系功能持续提升，政务服务综合标准化试点通过国家验收。推动法治政府建设，行政程序建设、行政复议等工作有效加强，自觉接受市人大及其常委会的法律监督、工作监督和市政协的民主监督，认真办好市人大代表议案、市政协建议案，完成人大代表建议、政协提案办理任务。深化廉洁政府建设，全面落实党风廉政建设责任制，认真学习中国共产党廉洁自律准则和纪律处分条例，加强政府系统惩治和预防腐败体系建设，驰而不息整治“四风”问题，实施公务用车制度改革。

　　各位代表，2015年各项目标任务的完成为“十二五”发展画上了圆满句号。过去五年，全市上下紧紧围绕“两个率先”，全力落实“八项工程”、实现“八个领先”，量质并举、创新转型、统筹发展、富民惠民取得重大成就。

　　（一）城市经济实力、产业竞争力、区域影响力同步并进，质量和效益持续提升。五年来，地区生产总值增长69.5%，在全国大中城市排名前移1位；一般公共预算收入增长115%，占GDP比重超过10%；进出口总额跃居全省第四。三次产业结构优化为5.8‥49.2‥45，服务业增加值占GDP比重提高7.5个百分点，高新技术产业、新兴产业产值占规模以上工业比重分别提高12.6个、11.5个百分点。全社会研发投入占GDP比重提高0.62个百分点，科技进步贡献率达60%，技能人才总数突破100万人，国家创新型试点城市、质量强市示范市和知识产权示范城市建设成效明显。单位GDP能耗下降21.5%，林木覆盖率提高5.5个百分点，绿色发展综合指数全省第一。

　　（二）沿江转型升级、沿海科学开发、陆海统筹发展取得历史性突破，生产力布局持续拓展。区域性综合交通枢纽加快形成，新建过江大通道2个，高速公路实现县县通，临海与沿江高等级公路实现环通，通江达海的铁路网加速形成，长江12.5米深水航道一期工程通航，江海组合大港初具雏形，内河高等级航道达126公里。陆海资源优化配置加快推进，沿江资源整合稳步实施，沿海“区镇合一”、海域综合管理等体制创新取得重要成果，连续24年保持耕地占补平衡。临港临海产业集群建设成效加快显现，五年实施370个市级以上重大项目，江苏LNG、恒力新材料、招商局海工等26个50亿元以上产业项目建成投产，沿海前沿区域生产总值和投资均突破千亿元。

　　（三）改革与开放相互促进，资源要素集聚能力持续增强。重点领域改革取得新突破，陆海统筹发展成为国家级综合改革试点，《陆海统筹发展综合配套改革试验区总体方案》由省委、省政府印发实施，六方面体制机制创新和100项重点改革任务扎实推进。对内对外开放迈出新步伐，民营经济、开放型经济、国有经济发展水平共同提升，个体、私营企业户数及注册资本居全省前列，引进来与走出去政策和体制先行先试成效明显，外资外贸结构优化，服务外包执行额年均增长50%以上，外经合作保持全省领先。载体和平台功能实现新提升，国家级开发园区达6个，国家一类开放口岸达5个，新增省级开发园区5个，各级开发园区转型升级深入推进，跨国跨江合作园区、沿江沿海特色园区协调发展，大宗商品交易、物流通关等“八类平台”加快形成。

　　（四）五级城乡空间体系初步构建，城乡统筹建设持续加强。中心城市竞争力不断增强，市区组团发展格局全面拉开，建成区面积五年增长64%，“102030”快速路网体系基本形成，城市功能、品质和形象显著提升。城镇综合开发水平持续提高，常住人口城镇化率达62.8%、五年提高6.8个百分点，5个县（市）中等城市框架加快构建，19个市级中心镇集聚能力明显增强，特色镇发展势头良好，规划布点村庄（新型社区）建设全面加强。县域经济竞相发展，各县（市）均跻身全国县域经济与县域基本竞争力百强行列，均成为全国科技进步先进县（市）、省文明城市。

　　（五）发展成果更多惠及全体人民，群众生活持续改善。社会建设“十大体系”加快构建，五年为民办实事215项（2015年45项实事完成情况见附件一）。城乡居民人均可支配收入分别增长69.2%、81.5%。覆盖全民的社会保障体系不断完善，城乡居民医保实现制度并轨，大病保险全面实施，城乡低保标准大幅提高，农村低收入人口整体实现5000元脱贫目标。多元化住房保障体系逐步健全。教育公平取得重要进展，5所高校升格更名，卫生、文化、体育等公共服务资源有效覆盖面稳步提高，养老服务形成“9073”格局，引江区域供水实现乡镇全覆盖。

　　（六）发展软环境进一步优化，城市美誉度持续提高。简政放权、放管结合、优化服务深入推进，全面取消非行政许可事项，取消、下放行政审批事项近400项，审批承诺期限压缩71%，群众满意度保持在98%以上，连续四年被台湾电电公会评为台商投资“极力推荐城市”。建成国家生态市，入选“中国最具幸福感城市”，获得“中欧绿色和智慧城市先行奖”，公众安全感全省第一。精神文明“南通现象”发扬光大，江海特色文化魅力进一步彰显，实现全国文明城市“三连冠”、双拥模范城“五连冠”。

　　各位代表，在“十二五”的发展实践中，我们始终坚持稳中求进、科学发展，主动适应、积极引领新常态，以经济建设为中心，一着不让稳增长，坚定不移转方式、调结构、惠民生，不断开创科学发展新局面；始终坚持抢抓机遇、增创优势，用足用好多重国家战略叠加效应，集聚新人才、壮大新产业、拓展新空间、建设新城市、构建新体制，培育体现区位特点、功能特色的新优势，全力提升发展竞争力；始终坚持解放思想、开拓创新，问题导向推进改革，旗帜鲜明鼓励创新，对标找差明目标、定举措，主动作为想在先、干在前，积极营造敢于突破、敢于争先的发展氛围；始终坚持实事求是、统筹协调，紧扣南通实际，全面推进“五位一体”建设，切实解决老百姓最关心、最直接、最现实的问题，努力创造彰显时代特征、顺应群众期盼的发展业绩。

　　各位代表，“十二五”发展成就的取得，是省委、省政府和市委正确领导，市人大、市政协监督支持，全市人民团结一致、艰苦奋斗的结果。我代表市人民政府，向全市人民，向人大代表、政协委员和离退休老同志，向市各民主党派、工商联、各人民团体和社会各界人士，向驻通人民解放军、武警官兵和公安干警以及部省驻通单位，向关心和支持南通发展的海内外朋友，向为南通发展付出心血的历任领导，致以崇高的敬意和衷心的感谢！

　　各位代表，“十二五”的实践也使我们清醒看到发展中的问题。经济转型面临考验，下行压力仍然较大，部分企业生产经营困难，供给侧、结构性和体制性矛盾并存，创新驱动力不强，南通还处于爬坡过坎阶段。发展短板仍然突出，均量水平、产业层次和开放度还不够高，城乡二元结构仍较明显，资源环境约束更紧，居民增收渠道不宽。社会建设任重道远，一些领域公共服务供给不足、资源配置不够均衡，人口老龄化程度加剧。维护社会安全稳定任务艰巨，新旧矛盾交织，基层基础相对薄弱，一些领域潜在风险不容忽视。政府自身建设仍待加强，职能转变还不到位，作风建设和反腐倡廉长效机制仍需完善。对此，我们将采取有效措施，着力提高发展的平衡性、协调性、包容性、可持续性。

　　二、“十三五”时期的目标任务

　　今后五年是南通率先全面建成小康社会的决胜阶段和积极探索开启基本实现现代化建设新征程的重要阶段，也是“迈上新台阶、建设新南通”的关键阶段。综观各方面形势，南通处于大有作为的重要战略机遇期和创新转型的黄金发展期，有基础有条件在抢抓国家战略叠加机遇中发展得好一些、快一点，在落实国家、省对南通发展定位中实现新的历史性跃升；国际金融危机影响仍将持续，中等收入阶段考验更加集中。我们必须准确把握发展趋势，有效应对各种挑战，奋力开拓各项工作新境界。根据《中共南通市委关于制定南通市国民经济和社会发展第十三个五年规划的建议》，市政府编制了《南通市国民经济和社会发展第十三个五年规划纲要（草案）》，现已发给各位代表，提请大会审议。

　　今后五年总体发展要求是：高举中国特色社会主义伟大旗帜，全面贯彻党的十八大和十八届三中、四中、五中全会精神，以马克思列宁主义、毛泽东思想、邓小平理论、“三个代表”重要思想、科学发展观为指导，深入贯彻习近平总书记系列重要讲话精神，以总书记视察江苏重要讲话精神为引领，紧紧围绕“四个全面”战略布局，坚持发展是第一要务，以提高发展质量和效益为中心，突出陆海统筹、江海联动，加快形成引领经济发展新常态的体制机制和发展方式，坚持稳中求进，深入落实“五个迈上新台阶”重点任务和省委“八项工程”，统筹推进经济建设、政治建设、文化建设、社会建设、生态文明建设，着力建设经济强、百姓富、环境美、社会文明程度高的新南通，率先全面建成小康社会，积极探索开启基本实现现代化建设新征程，谱写好中华民族伟大复兴中国梦的南通篇章。

　　今后五年主要发展目标是：科教进步、经济强盛。经济总量、均量、质量同步提升，地区生产总值增速快于全省1个百分点以上，服务业增加值占GDP比重50%左右，长三角北翼先进制造业和现代服务业基地初步建成，科教、产业、人才更加紧密结合。百姓富裕、人民幸福。居民收入增长和经济增长同步、劳动报酬提高和劳动生产率提高同步，完成新一轮脱贫任务，城乡居民人均可支配收入分别达5.5万元、2.65万元，民生投入增长快于经济增长，公共服务体系更加健全。空间集约、环境优美。港产城深度融合，城乡发展更加协调，户籍人口城镇化率65%左右，绿色发展能力显著增强，能源资源利用效率持续提高，集约高效的生产空间、宜居舒适的生活空间、天蓝水秀的生态空间基本形成。社会文明、风气良好。中国梦和社会主义核心价值观深入人心，人的素质全面提高，文化强市基本建成，文化产业增加值占GDP比重达6%，成为江苏有影响力的思想文化和道德风尚建设高地。制度优化、活力增强。治理体系和治理能力现代化取得重大进展，以经济领域为重点的各方面制度更加成熟、定型。

　　实现上述目标，必须牢记“两个率先”光荣使命，全面贯彻中央提出的五大发展理念和省委、省政府确定的七大发展战略，大力实施创新驱动和科教强市、陆海统筹江海联动、新型城镇化和城乡发展一体化、绿色发展、国际化和全面开放、民生优先六大战略，力争提前实现两个“翻一番”，努力让全面建成小康社会的水平更高、质量更优，在争当苏中新一轮发展“领头雁”、建设区域性综合交通枢纽（节点城市）、迈向长三角北翼经济中心上实现新突破，加快构建富有特色的科学发展、转型发展新格局。

　　（一）突出创新驱动，放大产业基础优势，加快构建提质、扩量、增效、升级相统一的平稳健康发展格局。大力推进制度、科技、文化等全方位创新，更多依靠创新驱动和发挥先发优势引领发展，更好增强科技、人才在全面创新中的核心作用，完善产业链、打造创新链、整合资源链、实现价值链，推动发展在中高速增长中迈向中高端水平，努力打造创新型城市和经济强市，加快形成以创新为主要动力的发展模式与经济体系。

　　（二）突出优江拓海，放大战略区位优势，加快构建陆海统筹、江海联动的集聚集约发展格局。抢抓国家推动形成沿海沿江沿线经济带等机遇，发挥南通与上海在国家战略布局中的区位互补优势，突出陆海统筹发展综合改革试点，以“多规合一”理念统筹全市域发展，以重大基础设施引领陆海空间调整，完善“五沿”生产力布局，努力打造陆海统筹发展试验区、江海联动开发示范区和江海交汇的现代化国际港口城市，加快形成发展导向明确、主体功能清晰、要素充分集聚的空间体系。

　　（三）突出统筹协调，放大城市功能优势，加快构建以城带乡、城乡一体的共同繁荣发展格局。更好统筹城乡经济社会协调发展、“四化”同步发展、硬实力与软实力整体发展，着力破除城乡二元结构，推动（沪）苏通经济圈建设，凸显在长三角城市群中的地位，努力打造协调型的长三角北翼中心城市，加快形成五级城乡空间体系和八个城乡发展一体化体系。

　　（四）突出绿色低碳，放大生态环境优势，加快构建资源节约利用、人与自然和谐的可持续发展格局。坚定不移走生产发展、生活富裕、生态良好的文明发展路子，坚持保护、建设、治理并重，构建科学合理的城市化格局、农业发展格局、生态安全格局、自然岸线格局，努力打造国内一流的生态宜居宜业城市，加快形成以绿色低碳循环为导向的生产生活方式和发展体系。

　　（五）突出开放提升，放大载体平台优势，加快构建内外互动、深层融合的全方位开放发展格局。深度融入国家新一轮对内对外开放大布局，充分发挥各级各类载体和平台作用，深入实施“六外”并举，全面提升引进来和走出去结合水平，努力打造新一轮国际先进产业转移的重要承接基地、“一带一路”海上战略合作支点、长江经济带北翼桥头堡和全面开放合作城市，加快形成东西双向拓展、国际国内市场联动的资源要素配置体系。

　　（六）突出民生优先，放大共建共享优势，加快构建人的全面发展、社会全面进步的发展格局。按照人人参与、人人尽力、人人享有的要求，坚持普惠性、保基本、均等化、可持续，紧紧围绕民生“七个更”，科学作出制度安排，深入推进社会建设，让广大群众对发展有更多的获得感，努力打造让市民自豪、人人向往、近悦远来的幸福城市，加快形成与全体人民共同发展相适应的社会进步体系。

　　各位代表，我们坚信，只要全市上下持续付出、埋头苦干，“强富美高”新南通一定能展现出现实的模样，南通人民的梦想一定是可以实现的！

　　三、2016年主要工作

　　2016年是实施“十三五”规划的开局之年，也是南通推进结构性改革、率先全面建成小康社会的攻坚之年。我们将根据中央、省、市经济工作会议部署，更好适应、引领新常态，认真落实宏观政策要稳、产业政策要准、微观政策要活、改革政策要实、社会政策要托底的要求，突出抓好“去产能、去库存、去杠杆、降成本、补短板”五大任务，统筹做好稳增长、调结构、惠民生、防风险各项工作，确保“十三五”发展开好局、起好步。

　　今年经济社会发展的主要预期目标是：地区生产总值增长9%左右；一般公共预算收入增长10%；固定资产投资增长12%左右；社会消费品零售总额增长10%左右；进出口总额保持增长；城镇和农村居民人均可支配收入增长与经济增长同步；居民消费价格涨幅3%左右；城镇登记失业率4%以内；全社会研发投入占GDP比重2.6%；节能减排完成省下达任务。

　　工作中，按照“十个更加注重”的要求，着重抓好十方面：

　　（一）保持稳增长和调结构平衡。培育发展新动力，构建产业新体系，实现更高质量更有效益更可持续的稳定增长。

　　推动先进制造业与现代服务业双轮驱动。完善政策措施，提高产业核心竞争力。做强做大主导产业。推动船舶及重装备等六大主导产业高端化发展，提升技术、质量、品牌和标准化水平，规模以上工业总产值增长9%。打造绿色、智慧建筑骨干企业和产业化基地。运用技术、市场、经济等手段化解过剩产能。壮大战略性新兴产业。围绕“3+4”重点领域，突破海洋工程、航天航空、生物医药、新材料等行业，新兴产业产值超过5000亿元。围绕集成电路、医学组织工程等成长性领域，建设一批特色产业基地。推进制造业服务化。以纺织服装、装备制造等为重点，推广定制化生产、柔性化制造、个性化服务模式。加快发展现代服务业。突出抓好集聚区建设、项目推进、楼宇经济、企业培育，服务业增加值占GDP比重达46%。加快发展生产性服务业，培育省级以上物流企业和特色园区，创建国家服务外包示范城市。壮大工业设计、供应链管理、电子商务等新兴服务业。

　　推动传统增长点与新兴增长点相互促进。坚持“互联网+”与“+互联网”并举，大力变革生产方式、服务模式。推广智能制造。实施企业互联网化提升、制造装备升级计划，发展智能工厂、智能车间，创建“两化融合”示范试点企业30家以上。提高船舶、机械等装备自动化成套能力，壮大工业机器人、3D打印等产业。发展网络经济。加快信息基础设施建设，全市宽带IP城域网出口带宽达2500G。支持依托互联网的商业模式和供应链、物流链创新，建设大数据和云计算中心、服务外包、动漫衍生等互联网产业园区，做大一批本土互联网企业。培育平台经济。建设互联网信息、交易、媒体、支付等服务平台，完善大宗商品交易、研发设计、检验检测等专业化平台，打造平台经济集聚区。

　　推动需求与供给协同发力。保持有效投入强度。实施总投资5050亿元的160个市级重大项目，推动产业改造升级、基础设施建设、新型城镇化、公共产品和服务供给等各类投资共同发力。发挥政府投资项目引导作用，带动各类资本参与重点领域投资。稳定和扩大出口。落实出口退税、进出口降费等措施，支持企业开拓国际市场，加快形成新的外贸增量。发展服务贸易、跨境贸易电子商务等新业态，培育外贸竞争新优势。充分挖掘消费潜力。壮大线上线下互动消费，发展快递业和消费金融，促进居民商业消费。提升旅游服务，丰富旅游产品，打造新兴休闲旅游目的地。建设智慧南通公共服务平台，实施教育、医疗、交通等29个智慧应用项目。培育健康、养老等消费热点，稳定房地产市场和消费。

　　（二）完善区域特色创新体系。聚焦实施《中国制造2025南通纲要》，加快建设创新型城市，全力打造江苏特色创新基地、特色产业基地和上海科创中心重要的产业化基地。

　　壮大创新创业主体。加快创新政策、资源、要素向“五自”企业集聚，高新技术企业新增100家、产业产值超过6800亿元。支持企业建立“一站两院三中心”，新建省级以上研发机构46家。落实加计抵扣、加速折旧等政策，引导企业增加创新投入。完善创业担保贷款和减税降费等措施，建设创业基地，支持科技人员、大学生、农民工等各类主体创新创业。整合创新项目、技术、人才、载体、资本等要素，培育一批优秀“创客”。

　　促进创新集聚集群发展。推动创新载体提升。对接苏南国家自主创新示范区，增强南通高新区、市北高新区、产业技术研究院等高端载体引领创新的能力。推动省级高新区功能开发，支持有条件的园区创建省级高新区。加快创新公共平台建设。整合财政资金支持种子产业发展，打造大型科学仪器设施共享平台和一批开放式的公共技术服务平台，加强产业技术创新联盟建设。支持“四众”空间发展。推动省级以上开发园区众创空间全覆盖，打造市级示范性众创空间，建设众包、众筹、众扶等平台。

　　建设创新人才高地。突出领军人才和团队，实施新一轮产业人才发展计划和“江海英才”计划，新增国家“千人计划”人才20名、省“双创”人才30名。推动各类人才协调发展，加强企业家队伍建设，新增高技能人才2万人。健全服务体系，完善创业激励、分类评价等机制，为各类人才提供专业化服务。

　　优化大众创业、万众创新环境。强化产学研协同创新，与知名高校、科研院所共建一批创新研发、技术转移机构，推动更多重大科技成果和合作项目在通产业化。发挥产业投资引导基金作用，继续发行“通创币”，大力引进创投和天使投资机构。深化国家质量强市示范市建设，加强知识产权保护和运用。

　　（三）强化陆海统筹江海联动开发。突出提升资源利用整体效益，打造沿江产业技术创新带、沿海创新创业走廊。

　　优化发展布局。深化沿江沿海规划“一张图”研究，完善港口运营管理机制，加快港产城融合发展。突出生态优先、绿色发展，加大沿江港区、岸线资源整合力度。以实施长江12.5米深水航道二期工程为契机，加快通海港区、如皋港区建设。促进沿海洋口、通州湾、吕四三大港区错位发展，推进深水航道、码头泊位建设。有序实施海门港等8个作业区开发。以沿海前沿区域重点镇为依托，推进12个功能区建设，打造沿海特色产业带、城镇带和风光带。高标准建设通州湾江海联动开发示范区，加大优质产业项目招引力度，加快培育科教城等特色园区和平台。

　　建设枢纽城市。完善综合交通体系，推进一批公铁水、江海河联运工程。实施铁路西站综合枢纽、沪通铁路平东货场等项目。支持沪通铁路（沪通长江大桥）、宁启铁路二期加快建设，争取盐通客专早日开工，做好通苏嘉城际、北沿江高铁前期工作。推进海启高速建设，开工建设锡通高速北连接线。开展长江口北支航道前期研究。实施兴东机场改扩建工程。

　　发展海洋经济。优化海洋产业布局，推进开发性金融促进海洋经济发展试点，争创省级海洋经济创新示范园区，海洋生产总值达1850亿元。做深做精沿海装备制造、现代物流、综合能源特色产业链，培育海洋生物、医药等新兴产业，壮大滨海旅游业，打造集水产品加工、交易、物流、休闲等于一体的渔港经济区。科学围垦和利用滩涂，创建省级滩涂综合开发试验区。

　　（四）推动改革举措落地见效。强化问题导向，突出重点领域，把握关键环节，加快把改革红利转化为发展动能。

　　着力抓好以陆海统筹发展综合改革为龙头的各级改革试点。创新陆海资源配置，编制实施陆海统筹发展土地利用规划，推进陆海空间置换和同一乡镇村庄建设用地布局调整等试点，扩大土地有偿使用范围，加强海域资源市场化配置，探索陆海资源统一交易。深化相对集中行政许可权改革，实施“一窗受理”和多图、多评联审等制度。稳步推进部门内部、跨部门跨行业和区域性综合执法体制改革。放大市场采购贸易效应，加快内外贸一体化。

　　积极推进供给侧结构性改革。推动财税、科技体制改革。完善全口径预算管理体系，放大财政政策激励效应。推广中关村等政策，健全技术创新市场导向、创新成果转化激励机制。加强金融创新和风险防范。引进和培育各类金融机构，建设市级专业金融集聚区。力争上市企业和“新三板”挂牌企业总数超100家，支持发展股权和债券融资，提高直接融资比重。深化离岸金融、跨境结算、个人境外投资、跨国公司资金集中运营等试点，鼓励发展融资租赁、融资担保。有效防范化解金融风险，优化政府性债务结构。健全现代市场体系。提升商品市场功能，发展土地、产权、金融、人才等重点市场，深化重点领域价格改革。

　　大力促进多种所有制经济竞相发展。释放民营经济活力。鼓励民营资本依法进入基础设施、金融、公共服务等领域，推进民营经济转型升级。深化国资国企改革。加快资产证券化、股权多元化、投资市场化，推进市属国有企业功能性重组、资产经营公司实体化改造，提高国有资产经营管理水平。优化实体经济发展环境。实施降低实体经济企业成本行动，采取改革、财税、金融、价格等手段，帮助企业降本增效、提高竞争力。

　　（五）全面扩大对内对外开放。坚定不移推进经济国际化，促进国际国内要素有序流动、资源高效配置、市场深度融合。

　　深入对接“一带一路”和长江经济带，提高交流合作水平。加大走出去力度。突出东盟、中南亚、非洲、中东欧等区域，拓展境外投资贸易和综合开发空间。积极参与国际产能与装备制造合作、基础设施互联互通，助推优势行业加快走出去，打造一批示范性境外园区（基地）。增强中奥、中意等国际合作园区功能。提升引进来质量。加大基地、园区和产业链招商力度，注册外资实际到账25亿美元，新引进亿元以上市外民资项目120个。大力招引新产业、新业态和功能性机构，提高现代服务业、新兴产业利用外资比重。推进一体化发展。突出长江经济带沿线重点城市，深化港口、产业分工与协作。发挥开发园区、口岸平台作用，增强物流、中转、贸易功能，提升服务长江流域能力。

　　强化配套服务上海，增创开放新优势。以南通综保区为龙头，加快复制上海自贸区体制机制创新经验，构建法治化、国际化、便利化的营商环境。完善投资贸易“单一窗口”，加快通关监管制度创新，推广金融和服务业改革开放新举措，推动与上海港口、机场开展实质性合作。增强苏通、锡通园区和市北高新等跨江合作园区承载能力，积极承接上海、苏南优质产业转移。

　　加强载体和平台建设，加快功能开发提升。推动国家级开发园区创新发展，提升承接信息、服务贸易、总部经济等高端业态的能力。促进省级开发园区特色发展，创建一批省级以上特色产业园区、创新型园区、知识产权园区，支持有条件的开发园区提档升级。鼓励重点开发园区建设贸易创新、展示展销、金融服务等平台。加快电子口岸互联互通，拓展一类开放口岸功能。

　　（六）推进国家现代农业示范区建设。加大强农惠农富农力度，加快农业结构调整和方式转变，推动农业现代化。

　　培育新型农业经营主体。积极发展家庭农场、专业大户、农（渔）业产业化龙头企业，开展农民合作社综合社建设试点，新增“全托管”经营主体500家，市级以上示范家庭农场30家、龙头企业30家。引导土地经营权规范有序流转，发展适度规模经营。加快建设新型职业农民队伍。推进供销合作社综合改革。

　　构建现代农业产业体系。编制实施农业产业空间布局规划，大力发展高效农业、生态农业、外向型农业，做大做强农业优势、特色产业，加快农业标准化、品牌化、专业化步伐。发展“一产接二连三”全产业链模式，建设一批现代农（渔）业园区、农产品加工集中区和休闲旅游农业精品景点。抓好“米袋子”和“菜篮子”，保障农产品供应。

　　增强农业发展支撑能力。加强农田水利建设和农业综合开发，提高农业机械化水平，新建高标准农田20万亩。加快农业科技创新，健全农业社会化服务体系。做好第三次全国农业普查。鼓励发展“互联网+”龙头企业、家庭农场及农产品众筹等生产营销模式，建设一批农业农村电子商务示范基地。

　　（七）加快新型城镇化和城乡发展一体化。落实中央城市工作“五大统筹”要求，优化城乡空间结构、人口布局和发展格局。

　　强化中心城市龙头作用。完善发展体系。深化城市总体规划修编和市区“十张网”规划研究，构建“一核四片区”发展布局。统筹新城区繁荣、老城区更新，实施“五山”资源整合，提升濠河景区形象，加快观音山新城、星岛水岸等片区开发，增强城市核心区功能。完善南通开发区、苏通园区等南部片区产业布局和配套设施。加强以市北新城为主体的北部片区功能开发。推进以通州城区、南通高新区为依托的中部片区南进西拓。推动以通州湾示范区为重点的东部片区加快发展。提升服务功能。推进507个城建项目，完成投资196亿元。拓展提升快速路网，实施42个骨干路网项目，以片区化理念推进支路网建设。深化城市轨道交通建设前期工作。推动公交优先发展。创建“海绵城市”，探索地下综合管廊试点，健全水电气和信息等服务体系。优化居住环境。抓好新一轮城市环境综合整治。制定棚户区改造三年行动计划，加快城中村、老小区综合改造和危房解危，强化小区物业管理。加强历史文化保护。建设一批道路绿化带和综合性公园、小游园，创建国家生态园林城市。

　　加强城镇规划建设和管理。推进规划创新。以“多规合一”的理念统筹城乡规划调整，做好城市开发边界、永久基本农田、生态红线划定工作。按照五级城乡空间体系，指导各地开展城市总体规划修编，完成镇村布局规划优化调整。提升城镇功能。支持5个县（市）强化片区发展、优化空间结构。推动国家和省新型城镇化改革试点镇、市级以上中心镇以及一批特色镇加快产城融合步伐。

　　加强城镇和农村交通、水利、污水处理等基础设施建设与管护，拓展电力、信息基础设施布局。强化综合治理。加快城市综合管理、环境综合整治向镇街延伸，深化村庄环境整治，提升农村环境“四位一体”长效管理水平。

　　创新城乡发展一体化体制机制。推进新型城镇化综合试点，深化户籍制度改革，完善成本分担、公共服务等机制，促进农业转移等人口落户城镇。支持有条件的地区推进城乡社会保障一体化。深化农村综合改革，基本完成土地承包经营权确权登记颁证，完善农村产权交易市场体系，审慎开展农村土地征收、集体经营性用地入市、宅基地制度等改革试点，创新农村金融产品和服务。

　　（八）打造生态宜居的美丽南通。坚持陆海生态环境统一管理，制定实施新一轮生态文明建设三年行动计划。

　　加强环境保护和建设。实施蓝天行动，落实扬尘管控、挥发性有机物治理、秸秆禁烧和综合利用等措施，PM2.5平均浓度降幅完成省下达任务。实施清水行动，开展新一轮水环境综合整治，推进重点流域上下游协同治理，加强海域综合利用管理以及饮用水源地保护和应急水源建设。实施绿地行动，抓好土壤、农业面源、固体废弃物污染防治，加大“两沿三园”绿化造林力度。实施重大生态建设工程，构建“六廊五圈多点”生态空间格局，提高环境基础设施建设和有效利用水平，依法打击环境违法行为。

　　促进循环低碳发展。推行企业循环式生产、产业循环式组合、园区循环式改造，实施一批清洁生产、资源综合利用示范工程，大力发展生态产业，全面推进省级以上开发园区创建生态园区。实行能源和水资源消耗、建设用地等总量、强度双控管理，实施50个重点减排项目和一批节能技改项目，健全重点用能单位能源管理体系。深化重点行业和化工园区专项整治。

　　健全生态文明制度体系。严格执行生态红线区域保护规划和主体功能区规划，落实生态红线和水环境区域补偿政策，推进排污权有偿使用和交易。完善环境治理政策，逐步建立覆盖所有固定污染源的企业排放许可制度，强化环保信用评级结果应用。建立生态文明绩效评价考核和责任追究制度。

　　（九）提高社会文明程度。以文化建设和社会治理为重点，为经济社会发展营造更好环境。

　　繁荣江海特色文化。培育和践行社会主义核心价值观，弘扬南通优秀传统文化，进一步推进“诚信南通”和公民思想道德建设。深入开展群众性精神文明创建活动，争创全国文明城市群。创建省级公共文化服务体系示范区，制定实施中心城区文化设施布局规划，推进镇（街道）、村（社区）综合文化服务中心建设。加强“书香南通”建设。扶持文艺精品创作。支持发展骨干文化企业和创意文化产业，培育新型文化业态，文化产业增加值占GDP比重达5%。打造一批重点文化和体育产业园区（基地），鼓励发展文化交易平台和专业市场。

　　加强和创新社会治理。全面推进依法治市，深入实施“七五”普法规划。提高社会治理法治化、精细化、科学化水平，规范县（市）、镇、村三级社会治理服务平台运行。增强城乡社区服务功能，健全社区减负长效机制，93%的城市社区、89%的农村社区达到省级和谐社区标准。积极培育社会组织，推动政社分开、政社合作、政社互评。强化基层民主建设，组织新一届村（居）委会选举。落实社会稳定风险评估制度，完善社会矛盾排查预警和调处化解综合机制，依法加强和改进信访工作。

　　保障公共安全。落实最严格的安全责任和管理制度，推进安全生产标准化建设，创建食品安全城市。以信息化为支撑加快建设立体化、现代化社会治安防控体系，严密防范、依法惩治违法犯罪活动。提高防灾减灾和应急管理能力。构建信息安全保障体系。深化军民融合发展，加强国防动员和后备力量建设，做好国家安全和双拥、民族、宗教、档案、保密等工作。

　　（十）不断增进民生福祉。认真贯彻就业优先和人口发展等战略，加大民生投入，办好十方面49项实事项目。

　　提高就业收入水平。实施更加积极的就业政策，完善就业服务体系，建设人力资源服务产业园，新增就业8万人。健全科学的工资水平决定、正常增长、支付保障机制，多渠道增加居民经营性、财产性收入。拓展农民增收空间，推进城乡低保一体化。着力缓解相对贫困，强化精准扶贫，防止因病返贫问题。

　　增强社会保障能力。推进“全民参保登记计划”，基本实现法定人员全覆盖。完善城乡居民基本养老保险制度，发展职业年金、企业年金、商业养老保险。提升大病统筹水平，深化医疗保险市级统筹和城乡统筹，健全可持续的筹资机制、报销比例调整机制。完善住房保障制度，实现市区动迁异地安置“先建后征”。健全社会救助体系，支持发展慈善、红十字、残疾人等事业。

　　扩大公共服务和产品供给。推进教育现代化。落实学前教育三年行动计划，加快义务教育学校标准化建设。推进普通高中优质特色发展，提升职业教育服务能力，加快实施高等教育国际合作项目。办好继续教育、特殊教育。实施乡村教师支持计划，推进现代学徒制等国家改革试点。放大体教结合优势，抓好足球改革发展。打造健康南通。深化医药卫生体制综合改革，完善现代医疗服务体系。提升各级医疗服务机构能力，加快市区卫生服务中心和社区卫生服务站标准化建设。加强医联体建设，推动医疗卫生服务下沉，完善分级诊疗制度。强化全科医生培养，优化基层卫生人才队伍结构。广泛开展全民健身活动。促进人口均衡发展。全面实施一对夫妇可生育两孩政策。推动养老服务格局向“9064”转变，促进医养融合对接，每千名老人养老床位数达33张。做好妇女、儿童、青少年、关心下一代等工作。

　　四、推动政府治理体系和治理能力现代化

　　时刻把责任扛在肩上、把纪律和规矩挺在前面、把忠诚干净担当记在心中，加快转变政府职能、提升政务效能、规范行政行为，扎实推进政府治理体系和治理能力建设。

　　（一）创新治理模式。着力构建适应供给侧改革的“放、管、服”体系。深化简政放权，全面落实行政审批、中介服务等领域“五个再砍掉一批”的要求，最大限度下放便民利民、基层亟需且能够承接的事项。坚持宽进严管，完善“3+N”事中事后监管体系，健全“双随机”等监管机制。持续优化服务，提升“12345”政府公共服务、基层便民服务平台功能，推广网上办理、代办代理、上门服务等方式，健全政府购买服务等制度。

　　（二）增强执行能力。坚持敢于负责、勇于担当、善于突破、勤于落实，弘扬马上就办、真抓实干作风，既鲜明支持主动作为，又严肃查处为官不为。完善重点经济工作综合考核和政府系统“创新奖”评选办法，加强绩效管理，营造鼓励首创、宽容失误的浓厚氛围。健全大督查格局和行政问责机制，加强专项督查，探索开展重大政策及工作落实第三方评估。

　　（三）深化依法行政。落实法治政府建设目标任务，稳步推进地方立法，规范制订政府规章。坚持依法、科学、民主决策，严格落实重大决策法定程序和跟踪反馈、责任追究等制度。自觉接受人大及其常委会的法律监督、工作监督和政协的民主监督，认真办好人大代表议案、建议和政协建议案、提案。重视社会监督和舆论监督，加强审计监督，深化政务公开，打造阳光政府。

　　（四）加强廉政建设。突出从严从实治政，认真贯彻中国共产党廉洁自律准则和纪律处分条例，严格执行中央八项规定和省、市委十项规定，坚决防止“四风”问题反弹。厉行勤俭节约，发扬艰苦奋斗作风。严格“三资”监管，完善公共资源交易平台，强化廉政风险防控。全面落实“两个责任”，健全政府系统惩防体系，严肃查处腐败案件。加强新常态下公务员教育管理，努力建设一支大局意识强、国际视野宽、工作作风实的队伍。

　　各位代表，新一轮发展的美好蓝图催人奋进。让我们更加紧密团结在以习近平同志为核心的党中央周围，在省委、省政府和市委的坚强领导下，同心同德、凝心聚力，为率先全面建成小康社会、积极探索开启基本实现现代化建设新征程而努力奋斗！
